# Challenges in the field of obstetric fistula

**DOI:** 10.1002/ijgo.13032

**Published:** 2020-01-13

**Authors:** Ajay Rane, Andrew Browning, Peter Majinge, Rachel Pope

**Affiliations:** ^1^ James Cook University Townsville Qld Australia; ^2^ FIGO (International Federation of Gynecology and Obstetrics) FIGO Fistula and Genital Trauma Committee London UK; ^3^ Maternity Africa Arusha Tanzania; ^4^ Comprehensive Community Based Rehabilitation in Tanzania (CCBRT) Dar es Salaam Tanzania; ^5^ Division of Global Women's Health Department of Obstetrics and Gynecology Baylor College of Medicine Houston TX USA

**Keywords:** End fistula, Obstetric fistula, UNFPA, Vesicovaginal fistula

## Abstract

Thirteen years after the last supplement on obstetric fistula, the authors challenge the progress achieved. Citing the ongoing need for a standardized classification system, uniform surgical training and certification, evaluation, follow‐up, and research, we emphasize the need for improved communication and coordination between government and nongovernment entities invested in ending obstetric fistula. Struck by the call by the United Nations to end obstetric fistula by 2030, we stress the need for increased and targeted funding of programs that are of the highest quality and impact.

## Abstract 2

A call to action on the greatest needs for ending obstetric fistula.

## 1 BACKGROUND

The authors reviewed the 2007 FIGO Supplement on the prevention and treatment of obstetric fistula^1^ and found it provocative reading. Thirteen years on, the list of “achievements” could be pages long; however, in reality, have we made any progress? Are we asking the difficult questions that we should be asking? Many of the needs addressed in the 2007 Supplement remain current issues in our field. These needs include a standardized classification system, uniform training and certification of surgeons, evaluation and follow‐up of outcomes, setting of research protocols, and the uniform setting of communication links.

Despite the many organizations and institutions working in the field of obstetric fistula, there is little collaboration and coordination of efforts. Leadership is needed to make greater strides to end obstetric fistula. Furthermore, the United Nations has set a goal to end obstetric fistula by 2030. The risks of such an ambitious goal are that it might encourage haste, unwarranted risk‐taking, inaccurate data, and pressure on countries and organizations to achieve a goal on “paper” that does not reflect reality. However, the advantage of such a lofty goal is that more attention is focused on the issue, intensifying resourcing and creating an opportunity to standardize the approach to ending fistula. This article provides an outline of where efforts need to be strengthened and where funding needs to correlate.

## 2 A STANDARDIZED CLASSIFICATION SYSTEM

A common language is needed for surgeons and health facilities to communicate surgical techniques and procedures for women with obstetric fistula. Standardized terminology is also needed to understand research conducted at diverse settings and to compare outcomes. This is a basic surgical tenet that is used in complex medical conditions such as gynecologic cancers and endometriosis. Obstetric fistula is a similarly complex, heterogeneous condition that language is needed to convey both the injury, as seen by any surgeon, and the repair that is required. Both FIGO and the International Society of Fistula Surgeons (ISOFS) are governing bodies that could assist in creating a uniform classification system. To begin the conversation on ending obstetric fistula, we need to speak the same language.

## 3 UNIFORM TRAINING AND CERTIFICATION

Some years back the term “fistula tourism” was coined.^2^ The concept and problems surrounding it still exist. Tourism happens when well‐meaning—but sometimes misguided—surgeons come from nonfistula‐affected countries to perform and teach fistula surgery in areas that are affected by obstetric fistula. They rapidly realize that, without adequate training and experience in the field, they are out of their depth and the local surgeons are too polite to say so. We have seen and heard of camps where visiting surgeons have come at great expense—promoting their good work back in their own countries—only to leave every woman they operate on still leaking; patients and hospital staff are left dismayed and the chance of the patient being cured is all the more remote owing to the failed operation. Worse still, there have been circumstances where the visiting teams have disrupted the national fistula programs to suit their own needs. Rogue camps and surgeons can cause more harm than good. When camps are set up in facilities not accustomed to providing care for fistula patients, the preoperative, intraoperative, and postoperative care is suboptimal. Fistula centers and experienced surgeons should be leading fistula repairs in facilities that are best equipped to offer care for this special patient population.

Additionally, in many countries there are local surgeons offering fistula repair who have not had adequate training. In some of the worst scenarios they charge women exorbitant costs to have surgery that is often unsuccessful. It is imperative that each country's ministry of health monitors the provision of fistula repairs to protect the rights of women with obstetric fistulae. Through FIGO and the Baylor College of Medicine/Texas Children's Hospital, training programs exist for surgeons and nonphysician clinicians in settings where there are no surgeons available. It would be prudent for each country's ministry of health to work with these entities to standardize training and certification for those offering surgical repair to women with obstetric fistula. Moreover, the multiple funding entities involved in the obstetric fistula arena should coordinate with ministries of health to fund only those individuals who are certified. Women with obstetric fistulae have been through enough trauma and should not be traumatized further by inexperienced and unskilled surgeons.

## 4 EVALUATION, FOLLOW‐UP, AND RESEARCH

Much of the research on obstetric fistula surrounds the lived experience and very little is robust research on surgical procedures. One of the biggest issues in our field is residual urethral incontinence—affecting approximately 33% of women with healed fistulae.^3^ The pathology is very different from women who have incontinence without history of fistula and limited research has taken place to help understand it. There have been incremental developments in preventing and treating the problem of ongoing incontinence despite fistula closure. However, we are still a long way off from fully understanding the problem; furthermore, the myriad approaches that have been developed to treat it make it clear that we still do not have the answer.

Women with obstetric fistulae have experienced a lack of access to the most basic of medical technologies: monitoring of labor and cesarean delivery. This injustice should not translate to a lack of access to innovation. There are biological and tissue engineering innovations in high‐resource settings that could enhance the quality of life for women with fistulae, especially in the area of urethral incontinence. However, as various techniques are developed, adequate follow‐up is needed to evaluate the long‐term outcomes. Without uniform evaluation and follow‐up of patients, including core outcomes, it is impossible to know whether we are making progress in any area.

Evidence suggests that in many countries there is an alarming increase in iatrogenic fistula patients.^4^, ^5^ Reasons for this are multiple and more research is needed. It is likely that improved surgical training and skill‐building are also needed. If this area is not addressed, it will only stymie the efforts to end fistula.

## 5 COMMUNICATION

Communication between governments and nongovernment entities invested in ending fistula is needed to train clinicians to prevent fistulae and repair those that have already occurred, generate evidence based on surgical techniques, and fuel innovations to improve the quality of life of women living with fistulae.

With the goal to end obstetric fistula by 2030, targeted and adequate funding is needed. In much of the international development community, the issue of “sustainability” is discussed. However, if obstetric fistula is a problem to be eradicated, our efforts do not need to be “sustainable,” but rather impactful and long standing. To repair the backlog of cases in a successful way, we need to fund the trained and certified surgeons so that the majority of cases are healed at the first attempt. Funding is needed to train additional surgeons to a standardized skill level so that more women can access high‐quality surgery. Follow‐up and evaluation will keep all entities on the right track. To prevent new cases, maternal health systems need ongoing support to bring basic obstetric care to all women.

Communication is needed to coordinate the various organizations and ministries of health to perform optimally. We have 10 years to reach the goal of ending obstetric fistula by 2030. Are we willing to do it?

## AUTHOR CONTRIBUTIONS

AR, AB, and RP conceived the concept for the manuscript. All authors contributed to drafting, editing, and finalizing the manuscript.

## CONFLICTS OF INTEREST

The authors have no conflicts of interest.
